# What methods are currently available for incorporating implementation considerations within the economic evaluation of health technologies? A scoping review

**DOI:** 10.1186/s12961-024-01220-9

**Published:** 2024-09-30

**Authors:** Robert Heggie, Kathleen Boyd, Hanin Kamaruzaman, Olivia Wu

**Affiliations:** Health Economics and Health Technology Assessment (HEHTA), Institute of Health and Wellbeing, University of Glasgow, Clarice Pears Building, 90 Byres Rd, Glasgow, G12 8TB UK

**Keywords:** Implementation, Economic evaluation, Health technology assessment

## Abstract

**Background:**

When clinically effective, cost-effective health interventions are not fully implemented in clinical practice, population health suffers. Economic factors are among the most commonly cited reasons for suboptimal implementation. Despite this, implementation and economic evaluation are not routinely performed in conjunction with one another. This review sought to identify and describe what methods are available for researchers to incorporate implementation within economic evaluation, how these methods differ, when they should be used, and where gaps remain.

**Methods:**

We conducted a scoping review using systematic methods. A pearl-growing approach was used to identify studies. References and citations were identified using Web of Science and Scopus. We included for review any study that contained terms relating to economic evaluation and a series of implementation-related terms in the title or abstract. The search was conducted and validated using two independent researchers.

**Results:**

Our review identified 42 unique studies that included a methodology for combining implementation and economic evaluation. The methods identified could be categorized into four broad themes: (i) policy cost–effectiveness approach (11 studies), (ii) value of information and value of implementation approach (16 studies), (iii) mixed methods approach (6 studies), and (iv) costing approach (9 studies). We identified a trend over time from methods that adopted the policy cost–effectiveness approach to methods that considered the trade-off between the value of information and value of implementation. More recently, mixed methods approaches to incorporate economic evaluation and implementation have been developed, alongside methods to define, measure and cost individual components of the implementation process for use in economic evaluation.

**Conclusion:**

Our review identified a range of methods currently available for researchers considering implementation alongside economic evaluation. There is no single method or tool that can incorporate all the relevant issues to fully incorporate implementation within an economic evaluation. Instead, there are a suite of tools available, each of which can be used to answer a specific question relating to implementation. Researchers, reimbursement agencies and national and local decision-makers need to consider how best to utilize these tools to improve implementation.

**Supplementary Information:**

The online version contains supplementary material available at 10.1186/s12961-024-01220-9.

## Background

Any health intervention is only as good as its implementation. Delayed or insufficient implementation of clinical and cost-effective health technologies leads to poorer health outcomes for patients and the suboptimal use of scarce resources for national health services. It is well documented that potentially valuable health interventions often fail to achieve widespread implementation [1]. There are many reasons why implementation may be suboptimal. However, one of the most commonly cited reasons is cost [2].

The value of a health technology is typically assessed in the UK using a cost–utility framework. Using this approach, the additional cost of a technology is compared with the additional utility obtained, where utility is most commonly measured as the quality-adjusted life years (QALYs) gained. If the cost per QALY gained is below an acceptable threshold, typically between £20,000–30,000 in the UK, the technology is considered cost-effective. However, the cost–utility framework was developed during a time when reimbursement agencies, such as the National Institute for Health and Care Excellence (NICE), typically assessed pharmaceutical interventions. With the growing use of companion diagnostics, medical devices and artificial intelligence (AI)-assisted decision-making, health interventions in a clinical setting are becoming increasingly complex. As such, it is necessary to consider how these technologies will be used in clinical practice.

The Medical Research Council (MRC) recently issued guidance that recommends that implementation should be considered alongside economic evaluation in the assessment of health technologies [3]. An update to the NICE guidance for technology appraisal in 2022 placed increased emphasis on additional costs associated with implementation, stating that an evaluation should include the full additional costs associated with introducing a technology [4]. However, there is currently a lack of formal guidance as to how implementation should be considered within the evaluation of a health technology.

Despite the lack of guidance in this area, progress is being made in the effort to consider implementation and economic evaluation alongside one another. In the field of implementation science, Roberts et al. found that, while the quantity of economic evaluations of implementation programmes remains modest, the quality of economic evaluations has improved over time [5]. Heggie et al. found that a small number of methods, such as stakeholder engagement and process evaluation, were being used to incorporate implementation within health technology assessments in the UK. However, implementation and economic evaluation were typically considered in isolation, rather than in conjunction [6].

To advance the use of methods that seek to incorporate implementation and economic evaluation within a single framework, this scoping review aims to map out all methods that are currently available for incorporating implementation within the economic evaluation of health technologies.

## Methods

We undertook a scoping review using systematic methods. A pearl-growing (also known as citation mining or snowballing) methodology was used to identify relevant studies [7, 8]. Compared with a traditional database searching approach, the pearl-growing approach has been shown to be more reliable for identifying studies from obscure or disparate sources [9, 10].

The pearl-growing approach involved the following six steps [11]. In step 1, we identified a specific study or article (the pearl). The choice of initial pearl was based upon consultation with researchers experienced in economic evaluation alongside implementation and on the prominence of this study within this field of research. Our choice of initial pearl was Fenwick, Claxton, and Sculpher’s article “The Value of Implementation and the Value of Information: Combined and Uneven Development” [12]. This study played a seminal role in the development of this area of research and is typically cited in any methodological study on the topic of implementation within economic evaluation. In step 2, we used Web of Science to identify and extract the citations and references of the initial pearl into a reference manager. In step 3, we applied predefined inclusion and exclusion criteria for studies to produce a set of studies suitable for inclusion in the review. Duplicate results were removed. In step 4, the citations and references of these studies were extracted to identify further pearls, and the inclusion/exclusion criteria were applied again. This process was repeated until the pearls retrieved no longer met our inclusion criteria. In step 5, a retrospective manual search of all of the pearls included for review was conducted to mitigate user or software errors. Finally, in step 6, we repeated steps 1–5 using our initial pearl on the Scopus database to ensure that all studies cited or referenced by our initial pearl were obtained. The process is illustrated in the additional material (Fig. A.1–4). Our study adhered to the Preferred Reporting Items for Systematic reviews and Meta-Analyses extension for Scoping Reviews (PRISMA-ScR) guidelines (Additional Material).

### Criteria for inclusion of studies

The four authors of this study undertook a brainstorming session to identify the key terms most commonly used within the literature on implementation within economic evaluation. We chose to include in our scoping review any studies that included the following terms in the title:

“implement*” OR “*reconfiguration*” OR “chang*” OR “*set-up*” OR “*uptake*” OR “*utilization*” OR “*capacity”.*

Any study that included these terms within the title was included for abstract review. Any study that included the following terms in the abstract was included for full manuscript review:

“economic*” AND (“implement*” OR “*reconfiguration*” OR “chang*” OR “*set-up*” OR “*uptake*” OR “*utilization*” OR “*capacity*”).

Following the full manuscript review, a study was included within our review if it described a methodology for incorporating implementation issues within the economic evaluation of a health technology. We included studies published over any time period. The initial review was undertaken in September 2022 (Additional Material Figs. 1, 2). The review was updated in March 2024 (Additional Material Figs. 3, 4).

### Criteria for exclusion of studies

No exclusions were made on the basis of participants, intervention, comparison or outcomes (PICO). As the purpose of this review was to identify currently available methodologies, no quality assessment of the identified studies was undertaken. Reviews and editorials were excluded. For practical reasons, non-English studies were excluded from the review. For the purpose of validation, one additional independent researcher applied the inclusion/exclusion criteria used in the pearl-growing process to the full set of studies identified in the search.

### Database search

We identified references and citations using the Web of Science and Scopus [13].

### Data extraction

All studies identified were exported to Endnote X9.3.3. The full manuscripts were reviewed to assess the content of the methodology utilized in the study. Content was assessed in terms of the approach used to consider implementation alongside economic evaluation. For the purpose of validation, one additional independent researcher assessed the content of each study to identify the approach to implementation utilized.

### Data synthesis and presentation

A content analysis was employed to identify and organize common themes (or approaches) in how implementation was incorporated alongside economic evaluation in the methodologies identified in our review [14]. We described what methods were available, how these methods differed from one another, when they should be used and where gaps remain. The extracted data are presented in tabular form (Table 1).

**Table 1:** Summary of the type of methodological approach used in each study identified in the review

	Methodological approach			
	Policy cost–effectiveness	Value of information and value of implementation	Mixed methods	Costing approach
Sculpher et al. [15]	**✓**			
Mason et al. [16]	**✓**			
Severens et al. [52]	**✓**			
Gandjour et al. [53]	**✓**			
Dijkstra et al. [54]	**✓**			
Wright et al. [55]	**✓**			
Fenwick et al. [12]		**✓**		
Hoomans et al. [56]	**✓**			
Hoomans et al. [57]	**✓**			
Hoomans et al. [58]		**✓**		
Hoomans et al. [59]	**✓**			
Wilan et al. [25]		**✓**		
Soeteman et al. [60]		**✓**		
Cheung et al. [61]			**✓**	
Fortney et al. [62]	**✓**			
Saldana et al. [37]			**✓**	
Walker et al. [18]		**✓**		
Andronis et al. [26]		**✓**		
Whyte et al. [63]		**✓**		
Faria et al. [64]		**✓**		
Grimm et al. [27]		**✓**		
Mewes et al. [65]		**✓**		
Hunter et al. [30]			**✓**	
Dopp et al. [29]			**✓**	
Cidav et al. [38]				**✓**
Eisman et al. [66]	**✓**			
Heggie et al. [67]		**✓**		
Johannesen et al. [19]		**✓**		
Wright et al. [20]		**✓**		
Clarke et al. [43]				**✓**
Dopp et al. [45]				**✓**
Eisman et al. [44]				**✓**
Salloum et al. [35]			**✓**	
Gold et al. [2]				**✓**
Heath et al. [68]		**✓**		
O’Leary et al. [31]			**✓**	
Wright et al. [69]		**✓**		
Cidav et al. [70]				**✓**
Pei et al. [28]		**✓**		
Smith et al. [71]				**✓**
Smith et al. [72]				**✓**
Smith et al. [36]				**✓**
*Frequency of method*	11	16	6	9

## Results

Our search identified 42 unique studies for inclusion in our review. On the basis of the studies identified in our review, four distinct approaches to considering implementation were identified: policy cost–effectiveness approach (11 studies), value of information (VoI) and value of implementation approach (16 studies), mixed methods approach (6 studies) and costing approach (9 studies). Each of the 42 studies identified fell into at least one of these categories; however, studies often overlapped a single category.

**Fig. 1:**
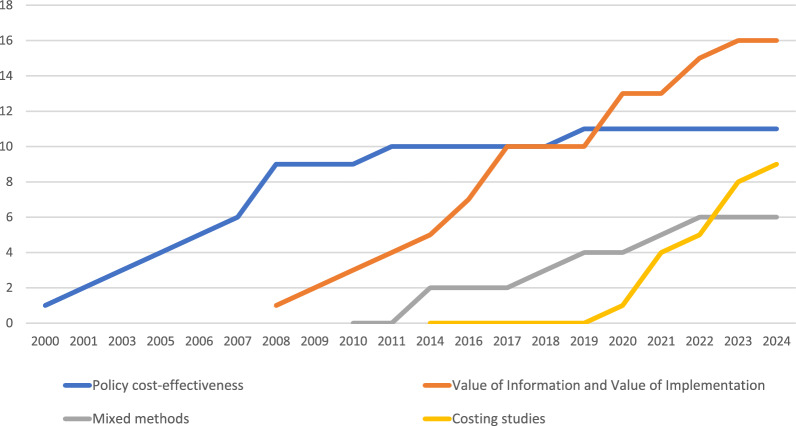
Number of articles published, by methodological approach, since 2000 to present

A clear trend is evident over time (Fig. 1). The majority of early methods in this area focused on policy cost–effectiveness – a comparative analysis of the implementation strategies. This evolved into methods designed to trade-off the value of further research (value of information) against the value of initiatives to increase the uptake (value of implementation) of a technology. These ideas were then used to develop tools for incorporating implementation issues within the design of studies. More recently, mixed methods approaches have been developed to incorporate implementation and economic evaluation alongside one another. Finally, methods have been developed to aid researchers in defining, measuring and costing individual stages of implementation for use in economic evaluation.

### What methods are available?

#### Policy cost–effectiveness approach

Approximately one quarter of the studies identified adopted the policy cost–effectiveness methodology (*n* = 11). Three of these studies used the simplest approach, developed by Sculpher et al. [15]. This approach involves treating the evaluation of an implementation strategy the same as any other new health intervention; that is, the costs and effects of the implementation strategy are compared incrementally with those of an alternative strategy or with no active implementation strategy. This is typically operationalized in a simple decision tree model.

The other eight studies utilizing the policy cost–effectiveness approach adopted the method of Mason et al. [16]. In contrast to the approach of Sculpher et al. [15], this approach combines both the costs and effects associated with a health intervention, in addition to the additional costs of implementation, to estimate an overall policy cost–effectiveness. This includes, for example, the additional costs of changing clinician behaviour and scaling this up to the total eligible patient population.

#### Value of information and value of implementation approach

The most common type of study identified utilized a method developed by Fenwick et al. [12] (*n* = 16). This approach built on the previous policy cost–effectiveness work of Sculpher [15] and Mason [16] and the work of Hoomans et al. [17], which focused on the decision of which evidence-based guidelines to adopt and how best to implement them. The methods discussed previously typically focused on the cost–effectiveness of specific implementation strategies. Fenwick et al. [12] were the first to consider the trade-off between the value of increasing implementation (that is, policies to improve uptake) compared with the value of increasing information (that is, further research to reduce decision uncertainty). They did this by considering four possible states of the world, where both information and implementation could either be perfect or at current levels, and the expected benefit of moving between states could be explicitly traded-off for decision-makers.

Based on the work of Hoomans et al. [17] and Fenwick et al. [12], a single value of implementation method was developed by Walker et al. [18]. This was distinct from the combined value of information and value of implementation method, for the context where further research is not considered, and the focus is on achieving a specific level of implementation. The value of implementation method, developed by Walker et al. [18] was then extended by Johannesen et al. [19] to subcategorize the total value of perfect implementation to estimate the relative value of eliminating different sources of suboptimal implementation [19].

All the methods for estimating the value of implementation identified thus far have assumed that the marginal costs and benefits associated with an intervention remain constant regardless of the level of uptake achieved. Wright et al. [20] extended the method developed by Walker et al. [18] to allow for the costs and benefits of an intervention to vary depending on the level of implementation. There are many reasons why costs and benefits could be expected to vary in practice, such as the need for initial capital outlays, capacity constraints or the existence of a learning curve for the delivery of a new procedure.

When reimbursement agencies consider a potentially valuable health technology with significant decision uncertainty, they may face the question of whether to approve the technology or recommend further research. This is a common consideration of the Scottish Medicines Consortium (SMC) and the National Institute for Health and Care Excellence (NICE) in the UK. Value of information and value of implementation methods can be used to inform these decisions. However, traditional VoI methods assume that the benefits of further information would be realized through full and immediate implementation. This is unlikely to be the case in healthcare provision [21–24].

The dynamic relationship between research and implementation was first considered by Fenwick et al. [12] in the form of the realizable expected value of perfect information (EVPI) – that is, the value of research that is realizable without actively undertaking strategies to increase implementation. This makes the simplifying assumption that information alone does not impact implementation. This assumption is unrealistic and is relaxed in the sensitivity analysis. Willan et al. built on this relationship between information and implementation to capture the impact this can have on the expected value of sample information (EVSI) and the cost of future trials [25]. Thus, they provide a method for informing research decisions and optimal sample size calculations, allowing for imperfect implementation. Andronis et al. developed a nonparametric approach for tackling the same problem, suggesting that the applicability of the method of Willan et al. is constrained by the fact that their approach assumes that outcomes (for example, net monetary benefit) are normally distributed [26].

Grimm et al. extended the previous work in this area by incorporating diffusion curves to model future implementation and by basing these curves on expert elicitation rather than assuming that implementation is solely a function of strength of evidence (as in previous methods) [27]. The authors found that the inclusion of diffusion curves had a significant impact on the value of further research, suggesting that it was inappropriate to assume static levels of implementation within value of implementation and information calculations. However, in this method, imperfect implementation is applied only to the value of implementation and not to the value of information. Thus, the benefit of reducing uncertainty is assumed to apply to all patients (including patients who do not stand to benefit from the information), potentially overestimating the true value of a trial [28]. Heath et al. [68] built on the work of Andronis et al. [26] by providing a nonparametric method to calculate the implementation-adjusted EVSI but without the unrealistic assumption that the speed of adoption and saturation level of the most cost-effective treatment are not related to future data. Additionally, Heath et al. [68] split out the impact of imperfect implementation on the value of implementation and value of information separately. Pei et al. [28] built on the work of Grimm et al. [27] by providing an approach for estimating the value of implementation and information separately but allowing imperfect implementation to affect not only the value of implementation but also the value of information (that is, since fewer people are able to benefit from that information).

#### Mixed methods approach

Implementation challenges are often not captured using the sort of quantitative methods discussed so far in this review. To address what they regard as the “qualitative residual”, Dopp et al. offered guidance on how to conduct a “mixed-methods” approach to economic evaluation in implementation research [29]. They do this by demonstrating how each item of the Consolidated Health Economic Evaluation Reporting Standards (CHEERS) checklist can be addressed from a mixed methods perspective – typically by complementing their quantitative analysis with qualitative insights. In the illustrative example they provide, data from qualitative findings were used to design cost surveys and inform key sources of uncertainty for sensitivity analysis. This allowed their economic evaluation to be tailored more exactly to its specific context.

Hunter et al. undertook an economic evaluation of the impact of the reconfiguration of stroke services in London and Manchester [30]. They used a difference-in-differences approach to estimate the change in cost and QALYs pre- and post-reconfiguration. However, in addition to presenting the results using the traditional metric of incremental cost per incremental QALY gained, they also used a Programme Budgeting and Marginal Analysis (PBMA) approach to report the results in terms of the number of QALYs gained, given a fixed budget and expected number of strokes per year for a hypothetical setting. The authors noted that, while a cost-per-QALY approach is more commonly utilized for economic evaluation, due to the influence of the National Institute for Health and Care Excellence (NICE), the incremental cost-per-QALY approach may not always be the most relevant to local decision-makers with a fixed budget who need to consider what return they can achieve for a given investment.

O’Leary et al. argue that current methods typically underestimate the resources required to implement complex interventions [31]. Building further on the mixed methods approach to economic evaluation and implementation, O’Leary et al. [31] suggest the use of the exploration, preparation, implementation, and sustainment (EPIS) method [32] as a vehicle for bringing in a range of tools necessary to conduct a full economic evaluation of complex interventions. The EPIS method is a conceptual model that highlights four key stages of implementation. On the basis of these four stages, O’Leary et al. [31] suggested a range of existing methods for data collection and analysis that are relevant from a health economic perspective. For example, stakeholder interviews within the exploration phase to identify their readiness to adopt a new intervention, and to identify likely barriers and facilitators. In the implementation phase, the use of simulation methods were used to compare the expected outcomes in the local context with those of the overall population, with the aim of identifying potential equity issues.

#### Costing approach

A budget impact analysis aims to estimate the total financial impact on a specific budget holder resulting from the implementation of a health technology [33]. Budget impact analyses are increasingly required by reimbursement agencies, alongside the traditional cost–effectiveness analyses [34]. Smith et al. [71, 72] highlighted the importance of budget impact analyses in the context of a tobacco use treatment programme. While these programmes are often shown to be good value for money, based on a cost–effectiveness analysis [35], the intervention must also be affordable within an organization’s available budget. In this context, a budget impact analysis can provide information to decision-makers on whether and how to implement an intervention [36].

Saldana et al. suggested that one reason that implementation costs are not routinely considered alongside the evaluation of health interventions is that there is a lack of standardized instruments for measuring implementation costs [37]. This may make it difficult for decision-makers to compare implementation costs across multiple potential health interventions. To this end, they developed a tool that maps costs on eight prespecified implementation stages of a foster care program, which allows for a cost comparison of implementation strategies. While this tool was developed for use in a foster care programme, it could easily be adapted for use in the evaluation of other health interventions.

Building on the work of Saldana et al. [37], Cidav et al. [38] developed a more general method that combined a time-driven activity-based microcosting (TDABC) method with the Proctor et al. [39] method for reporting standards in implementation research. The result is a method that allows the researcher to define “who (personnel completing the task) does what (specific activities performed), when (timing), and how often (the frequency, intensity and/or duration of the activity)” alongside Proctor’s guidance for the naming, defining and conducting of implementation strategies [40]. Together, this provides a tool for researchers to estimate resource use and cost for both a complete implementation strategy and for the distinct stages involved. These data can then be used to form the basis of budget impact analysis or to inform the economic evaluation of implementation strategies, where there may be a range of alternative implementation strategies available, each with their own associated costs and benefits.

The choice of which costs to include, for whom these costs are relevant, and over which time horizon is the focus of Gold et al. [2]. They argue that, over a longer time horizon, all costs are variable. However, over a short time horizon, it becomes important to distinguish between fixed and variable costs – something that is typically not observed in economic evaluations of health technologies. This is necessary because the costs and benefits of an intervention may accrue to different stakeholders if significant upfront investment is required at an early stage of implementation.

Major systems change (MSC) involves the reorganization or reconfiguring of healthcare services, typically in the form of a centralization of services, with a view to improving outcomes through greater specialization. Economies of scale may mean that this can be achieved at a comparable or reduced cost. However, quality economic evaluations that incorporate the implementation cost associated with MSC are lacking [41, 42]. Clarke et al. used the reorganization of cancer services in London as a case study to develop a method for costing the process of MSC [43]. Similar to that of Cidav et al. [38], this approach involves the specification of key stages in the implementation process. However, the evaluation perspective is also important when considering the implementation cost. To this end, Clarke et al. [13] go one step further and provide guidance on which implementation costs will be relevant for which perspective – provider, payer or national. These data can then be used to inform the economic evaluation of major system changes from the perspective of the relevant decision-maker.

In contrast with traditional economic evaluations, where the perspective is typically that of a healthcare system or society, Eisman et al. argue that the perspective adopted when considering implementation should be that of whichever stakeholder(s) or decision-maker(s) will be responsible for implementing the technology [44]. However, at present, many economic evaluations of implementation strategies fail to report costs from the perspective of multiple stakeholders. Incorporating such a perspective would help coordinate priorities among stakeholders and ensure that costs and benefits are distributed in such a way as to incentivize cooperation among stakeholders. To this end, Eisman provides guidance on how to incorporate multilevel stakeholder economic perspectives when implementing a health technology. This includes the preparation step, which involves simply identifying all key stakeholders; knowledge exploration, which involves discussing costs and priorities among stakeholders; and determining which strategies can produce win‒win scenarios among stakeholders.

Dopp et al. highlighted that there is little guidance on how to use evidence from economic evaluation to implement evidence-based practices [45]. They suggest that one approach is to use evidence from economic evaluation to develop bespoke financial strategies for implementation. To provide context, the authors highlight the so-called wrong pockets problem – that is, that the costs and benefits of health technologies may accrue to different stakeholders or in different sectors at different times. This creates the challenge of determining who should pay for the implementation of the intervention. In this environment, bespoke financial initiatives, tailored to the healthcare context, can overcome this barrier. The authors provide an example of a behavioural intervention to prevent detention and incarceration among youths. An economic evaluation estimated a return of US $3 to society for every $1 spent within 2 years post-treatment. However, the upfront cost of implementing this intervention – approximately $8000–13,000 per treatment – created a barrier to implementation. In a strategy known as pay-for-success, private investors interested in social impact were recruited to invest the initial capital required for fund implementation. Investors were then paid when measurable implementation outcomes were achieved.

### How do the methods differ from one another?

The main difference in the range of methods identified in this review is the purpose for which they were developed. While they all focus on the issue of implementation, four main approaches were identified – (i) the policy cost–effectiveness approach, (ii) value information and value of implementation approach, (iii) the mixed methods approach and (iv) the costing study approach. There are two distinct approaches for considering policy cost–effectiveness. The simplest approach, based on the work of Sculpher et al. [15] involves a comparative economic evaluation of the costs and effects (for example, QALYs, quality improvement, etc.) associated with implementing, or increasing uptake of, a health technology. This can take the form of a simple decision tree with the costs and effects of an implementation strategy compared with an alternative implementation strategy or no further implementation. This approach is methodologically straightforward. The challenge here is quantifying the cost and effect associated with each strategy. Tools for calculating the costs associated with implementation are available and have been highlighted in this review. Similarly, tools are available for estimating the health benefits of increased implementation. However, generating these data would represent an additional task in addition to the comparative evaluation of the overall impact of the implementation strategies. Therefore, while methodologically simple to employ, the data required to undertake such an analysis may be difficult and time-consuming to obtain. However, such analyses could be undertaken on the basis of assumptions and expert opinion – particularly for the purpose of determining thresholds where further implementation would (or would not) be likely to be considered worthwhile.

The second approach to considering policy cost–effectiveness, based on the work of Mason et al. [16], involves incorporating the cost of changing a physician’s behaviour (for example, the cost of implementing change per practice) in addition to the treatment cost–effectiveness (costs and effects per patient) of a health technology. This approach can be considered an extension of the approach of Sculpher et al. [15], which, rather than considering the process of heath technology evaluation and implementation strategy evaluation separately, combines the two concepts to derive an overall policy cost–effectiveness for a health technology.

The main distinction among methods identified in this review is whether or not implementation is the sole purpose of the analysis or whether this is a trade-off against the value of further research. Where implementation is the focus, the Walker et al. [18] approach is the most commonly used. Where the trade-off between information and implementation is the focus, the Fenwick et al. [12] approach is most commonly used. However, both methodologies have subsequently been further developed. Some methods to consider implementation in study design, which continue in the tradition of Fenwick et al. [12] focus on the interaction between information and implementation and the implications this can have for realizable EVPI (for example, the actual EVPI, given imperfect implementation), the cost of further research and the optimal sample size.

Value of information and implementation methods tend to require either a lot of data and/or a lot of assumptions. This is because we require estimates for parameters such as prevalence of the condition and lifespan of technology, alongside knowledge of relevant implementation strategies and costs. They also require a decision analytic model which can combine this evidence and undertake probabilistic sensitivity analysis.

Building on the methods already described, and on the frameworks already available in the field of implementation science, work has begun to incorporate both economic evaluation and implementation into a single framework [29, 31]. However, these methods are still relatively recent, and uptake of these methods remains to be seen. The data requirements of such methods will be higher than that of a standard economic evaluation. However, the benefit of these methods in terms of achieving implementation may justify the additional effort.

From a methodological perspective, the costing approaches of Cidav et al. [38] and Clarke et al. [43] are similar – they both seek to break down the implementation process into identifiable components, each of which can then be measured and valued for the purpose of inclusion in a full economic evaluation. The main difference between these tools is the purpose for which they would be used – the former for the evaluation of the implementation of a health intervention and the latter for the evaluation of major system changes. Costing methods tend to require a lot of data on the inputs (staff, setting, time, etc.) required to implement a programme. Such an estimate can be obtained using top-down approaches (for example, national unit costs) in some instances. However, to implement a new programme, detailed microcosting will often be required. This is a much more labour-intensive and time-consuming task.

### What gaps exist in the methods currently available?

There is no single method or tool that can incorporate all the relevant issues to fully incorporate implementation within an economic evaluation. Instead, there are a suite of tools available, each of which can be used to answer a specific question relating to implementation.

Current methods for considering implementation alongside economic evaluation typically focus on the value of increasing the uptake of a health technology and how this is compared with other objectives, such as further research. This assumes that we have a well-defined health technology that is ready to scale as required. However, prior to this step, it is first necessary to define how a health technology will be implemented. Many issues that were not identified or tested in clinical trials of health technology may pose challenges to its implementation in routine practice. For example, there may be differences relating to the clinical pathway for patients, modes of delivery, setup and training costs or any other aspect of how the technology is delivered in practice.

Although tools are available for identifying these issues within the trial setting – for example, qualitative methods – how these tools should be combined with economic evaluation tools is less clear. Dopp et al. [29] provide a first step in tackling this challenge with their guidance for mixed methods economic evaluations. However, our review did not identify any studies that used this guidance to date. No other methods for combining qualitative and quantitative data in the economic evaluation of implementation were identified.

## Discussion

To the best of our knowledge, there are currently no reviews of methods available for considering implementation alongside economic evaluation. Roberts et al. conducted a review of the use of economic evaluation methods in implementation studies [5]. They found that economic evaluation was not commonly applied within implementation studies. Furthermore, they highlighted that economic evaluations were typically conducted post-implementation, using retrospective data. This implies that economic evaluation did not play an important role in decision-making regarding implementation strategies.

Our review identified guidance for a mixed method approach to economic evaluation that incorporates implementation issues [29]. However, our review did not identify any examples of this approach used in practice. This may partly be explained by the recency of this guidance. However, further guidance will likely be necessary to describe how to combine qualitative and quantitative data in the economic evaluation of implementation. For example, how should we use qualitative data to inform our sensitivity and scenario analyses? What should we do when qualitative and quantitative findings are in conflict? How can qualitative data broaden our understanding of patient “value” in economic evaluation? And how would these results be used by decision-makers?

Methods identified in this review typically sought to estimate the value of implementation using the QALY outcome as the measure of benefit. However, the benefit of competing health interventions is not always sufficiently captured within a QALY outcome – either because the QALY is not feasible to capture or is not relevant in this context. Further research is necessary to develop methods for considering the importance of implementation in the context of a complex intervention, where multiple outcomes may be relevant to different stakeholders. Multicriteria decision analysis (MCDA) and discrete choice experiments (DCEs) provide tools whereby multiple outcomes can be traded off and valued for the purpose of healthcare decision-making. However, further guidance into how these methods should be used in economic evaluation is required [46, 47]. To date, these tools have not been utilized in the economic evaluation of implementation.

A strength and limitation of this review was the decision to categorize methods for incorporating implementation into discrete groups. The four categorizations chosen – (i) policy cost–effectiveness approach, (ii) value of information and value of implementation approach, (iii) mixed methods approach and (iv) costing approach – were based on this study’s authors’ judgement. We acknowledge that some methods may overlap categories or that additional categories may have been used by other researchers. However, we felt that such classifications were necessary to bring structure to the literature, which is at present disparate and difficult to navigate.

It is possible that an alternative choice of initial pearl would have led to a different final set of studies obtained. However, given that for a relevant study not to be captured within the review, it would need to not have been referenced or cited in any of the most referenced and cited studies in that area, it is unlikely that this process would fail to identify many relevant studies.

**Fig. 2:**
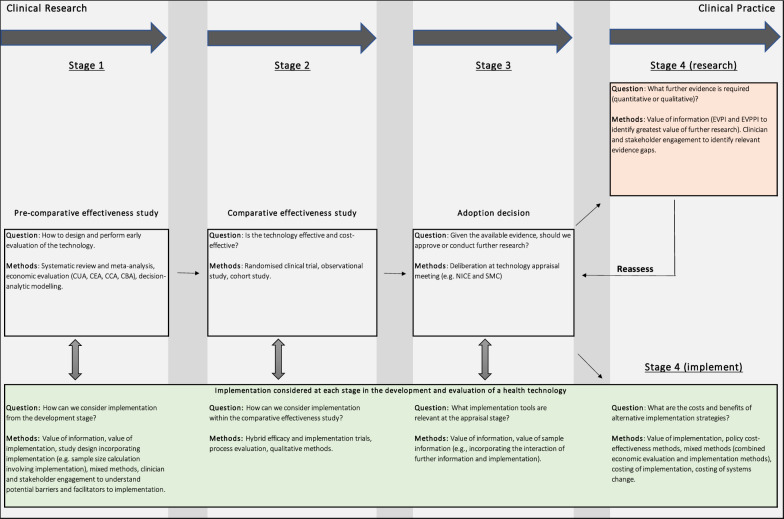
Conceptual model illustrating where methods identified in this review may contribute to the process of HTA

On the basis of the findings of our review, we can summarize the methods available for incorporating implementation within economic evaluation, alongside the standard methods of health technology assessment (HTA), in a conceptual model that suggests where these methods may be most relevant for the development, evaluation and implementation of a health technology (Fig. 2).

Stage 1 of the model describes the pre-comparative effectiveness study stage, where the focus is on the development and early evaluation of a health technology. At this stage, where the evidence base for a health technology is still under development, value of information [48] and value of implementation methods [18] can be used to identify key areas of uncertainty and inform study design (such as sample size calculation methods that incorporate implementation [25]). Engagement with clinicians and other stakeholders at this stage can help identify barriers and facilitators to implementation, inform and validate technology development and modelling requirements [49].

Stage 2 involves the assessment of clinical and cost–effectiveness data. In addition to the standard methods of clinical trials, observational studies and economic evaluation, methods involving hybrid effectiveness–implementation study design [50] and process evaluation [51] may also be appropriate.

Stage 3 represents the technology appraisal stage of the health technology assessment process. At this stage, the central question may be whether to approve the technology on the basis of current clinical and economic evidence or whether to recommend further research to reduce decision uncertainty. The Fenwick et al. method for considering the trade-off between investing in uptake and further research is particularly relevant at this stage [12].

Following this decision, the conceptual model focuses on the decision problem of implementation or further research. However, it should be noted that, as highlighted in the review, these two decision problems are not necessarily distinct and may interact with one another.

If the decision was made at stage 3 to undertake further research, stage 4 (research) involves the consideration of what sort of additional evidence is needed. Value of information methods (such as the expected value of perfect, partial and sample information) will be relevant. These analyses can be informed or supplemented with qualitative data obtained from clinician and stakeholder engagement. Once further research evidence is obtained, there is an option to return to stage 3 of the model and reassess whether to proceed with implementation or whether further research is still required to reduce decision uncertainty.

If the decision was made at stage 3 to implement, in stage 4 (implement), we can use the value of implementation, policy cost–effectiveness and costing methods to estimate the costs and consequences associated with efforts to increase the implementation of the technology.

It is important that economic evaluation and implementation be considered alongside one another when evaluating a health intervention. Decision-makers need to know not only the costs and benefits associated with a health intervention but also the challenges associated with its implementation. Future research should bring together experts from economic evaluation and implementation science, alongside representatives from health research funders, regulatory agencies and decision-makers, to develop formal guidance as to how implementation can be incorporated within the economic evaluation of health technologies.

However, as this review has demonstrated, methods are already available. Therefore, in addition to developing new methods, health economists and implementation scientists should work together to implement current methods for incorporating implementation within economic evaluation and increase the likelihood that promising health technologies are implemented in a timely manner.

## Conclusion

Our review has shown that a range of methods are currently available for researchers considering implementation alongside economic evaluation. While further research will be required to develop these methods, better coordination is also required among national reimbursement agencies and both national and local decision-makers to create an environment in which this type of research is both sought and utilized in decision-making. This is necessary to ensure that the costs and benefits of a health intervention are distributed fairly and that incentives are aligned among multiple stakeholders.

## Supplementary Information

Additional file 1: Fig. A.1. Flow diagram of the pearl-growing literature review in the Web of Science. Flow diagram depicting the pearl-growing search strategy process with reasons for study exclusion.

Additional file 2: Fig. A.2. Flow diagram of the pearl-growing literature review in Scopus. Flow diagram depicting the pearl-growing search strategy process with reasons for study exclusion.

Additional file 3: Fig. A.3. Flow diagram of the pearl-growing literature review in the Web of Science (updated). Flow diagram depicting the pearl-growing search strategy process with reasons for study exclusion.

Additional file 4: Fig. A.4. Flow diagram of the pearl-growing literature review in Scopus (updated). Flow diagram depicting the pearl-growing search strategy process with reasons for study exclusion.

## Data Availability

All data generated or analysed during this study are included in this published article (and its supplementary information files).
