# Supplementary figures and images for: What methods are currently available for incorporating implementation considerations within the economic evaluation of health technologies? A scoping review
Source: Health Res Policy Syst. 2024 Sep 30;22:134. doi: 10.1186/s12961-024-01220-9 (PMC11441006; doi:10.1186/s12961-024-01220-9)

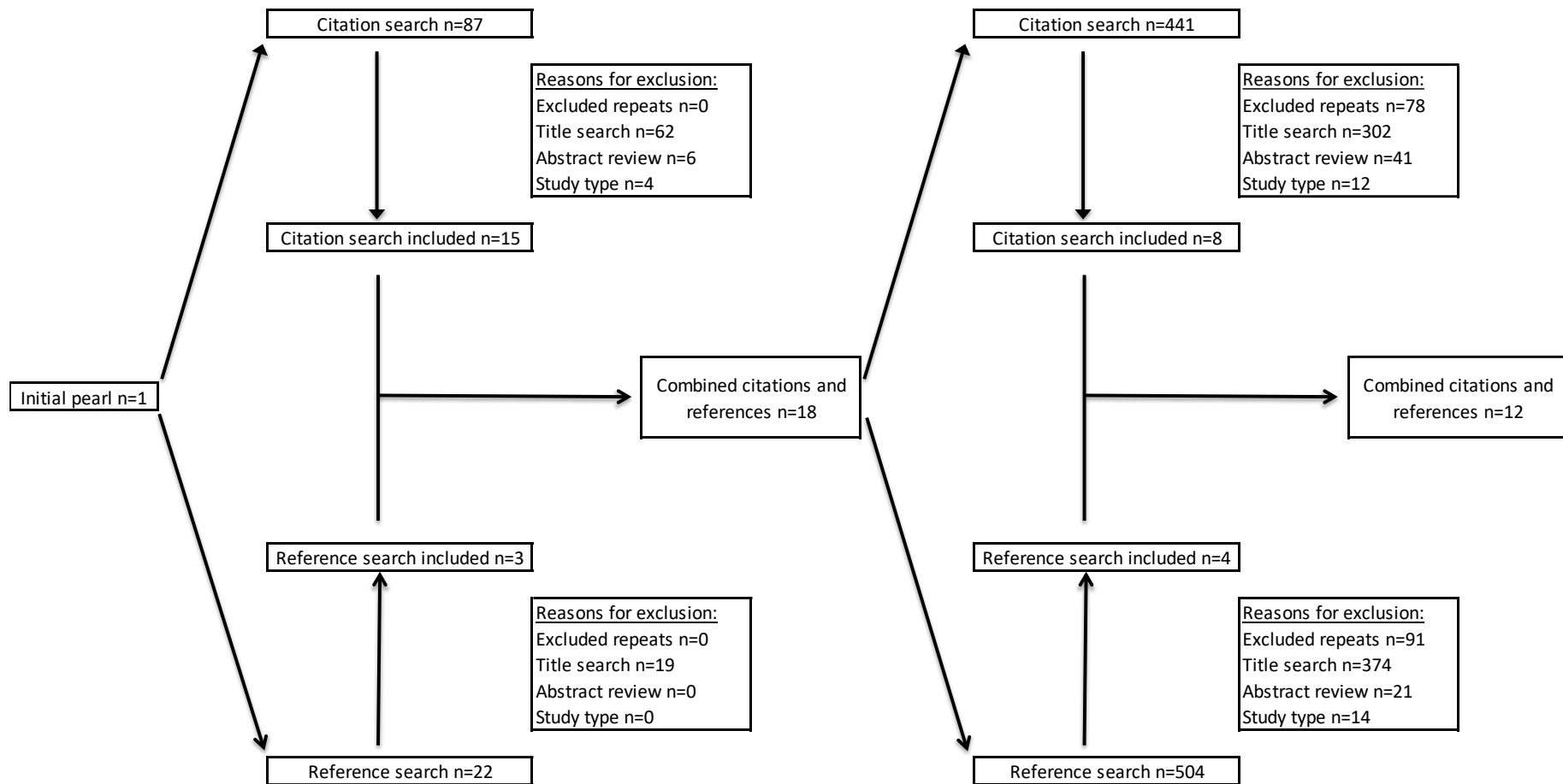

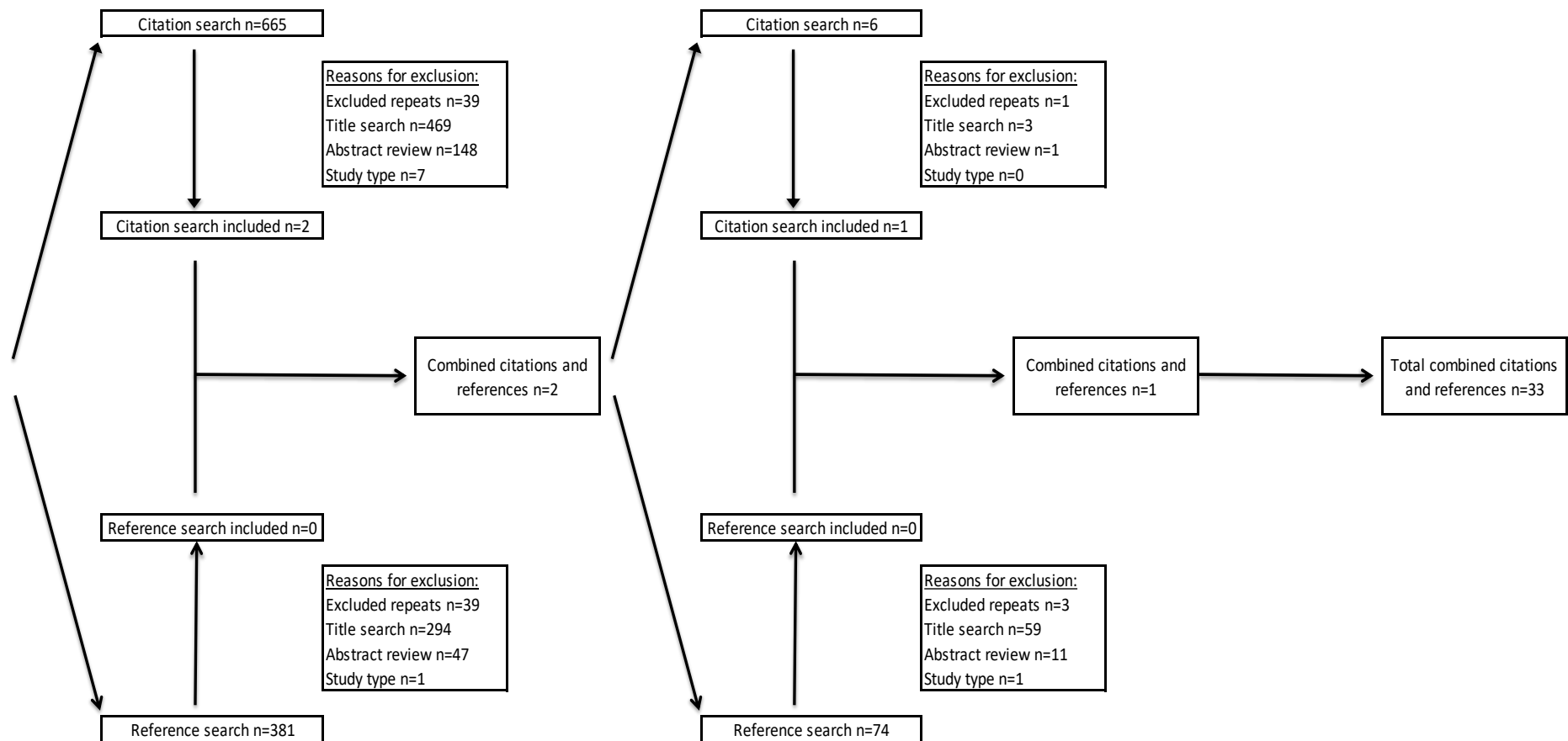

Figure A. 1: Flow Diagram of Pearl Growing Literature Review in Web of Science

Supplement: Supplementary file 1 — Additional file 1: Fig. A.1. Flow diagram of the pearl-growing literature review in the Web of Science. Flow diagram depicting the pearl-growing search strategy process with reasons for study exclusion. [file 12961_2024_1220_MOESM1_ESM.pdf]

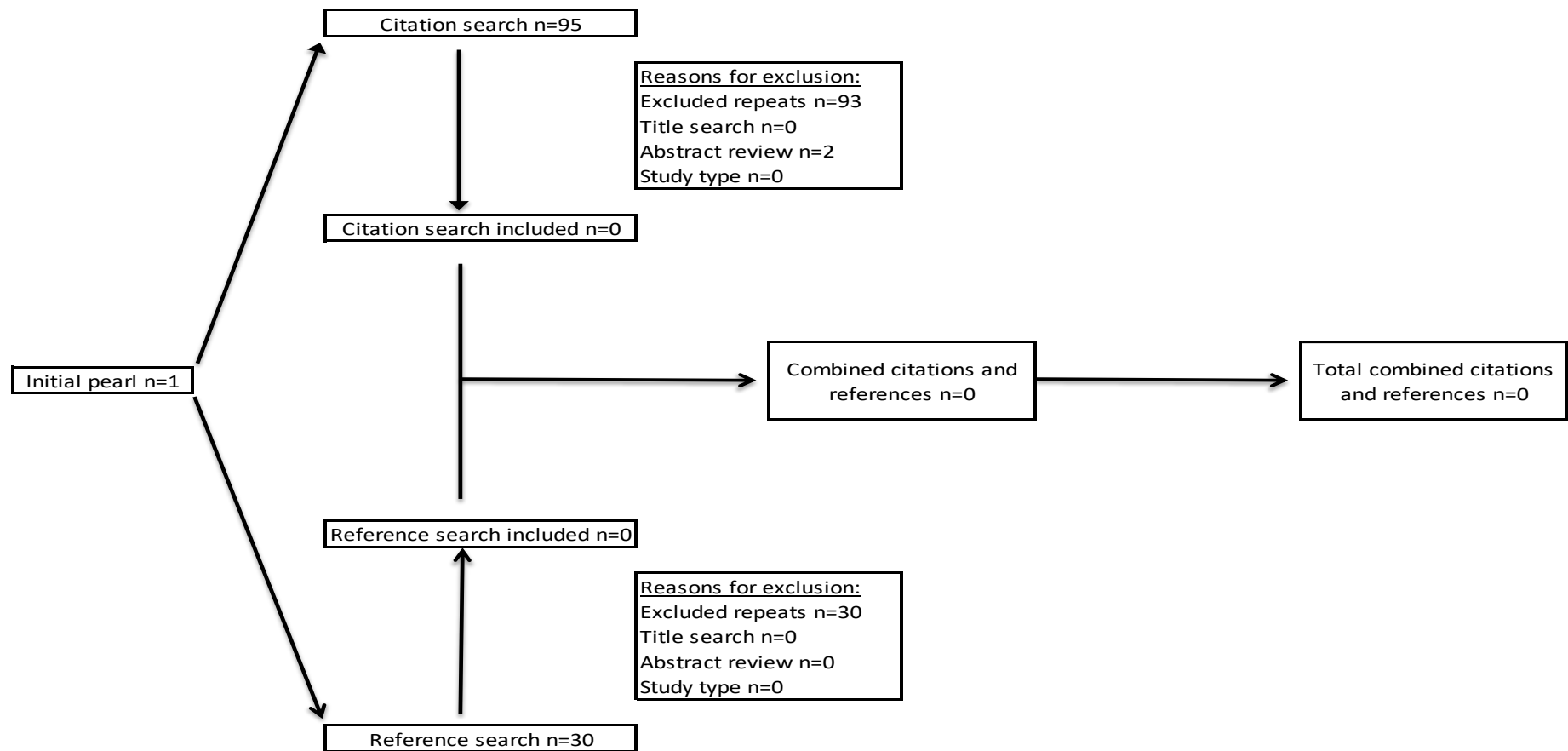

Figure A. 2: Flow diagram of the Pearl Growing Literature Review in SCOPUS

Supplement: Supplementary file 2 — Additional file 2: Fig. A.2. Flow diagram of the pearl-growing literature review in Scopus. Flow diagram depicting the pearl-growing search strategy process with reasons for study exclusion. [file 12961_2024_1220_MOESM2_ESM.pdf]

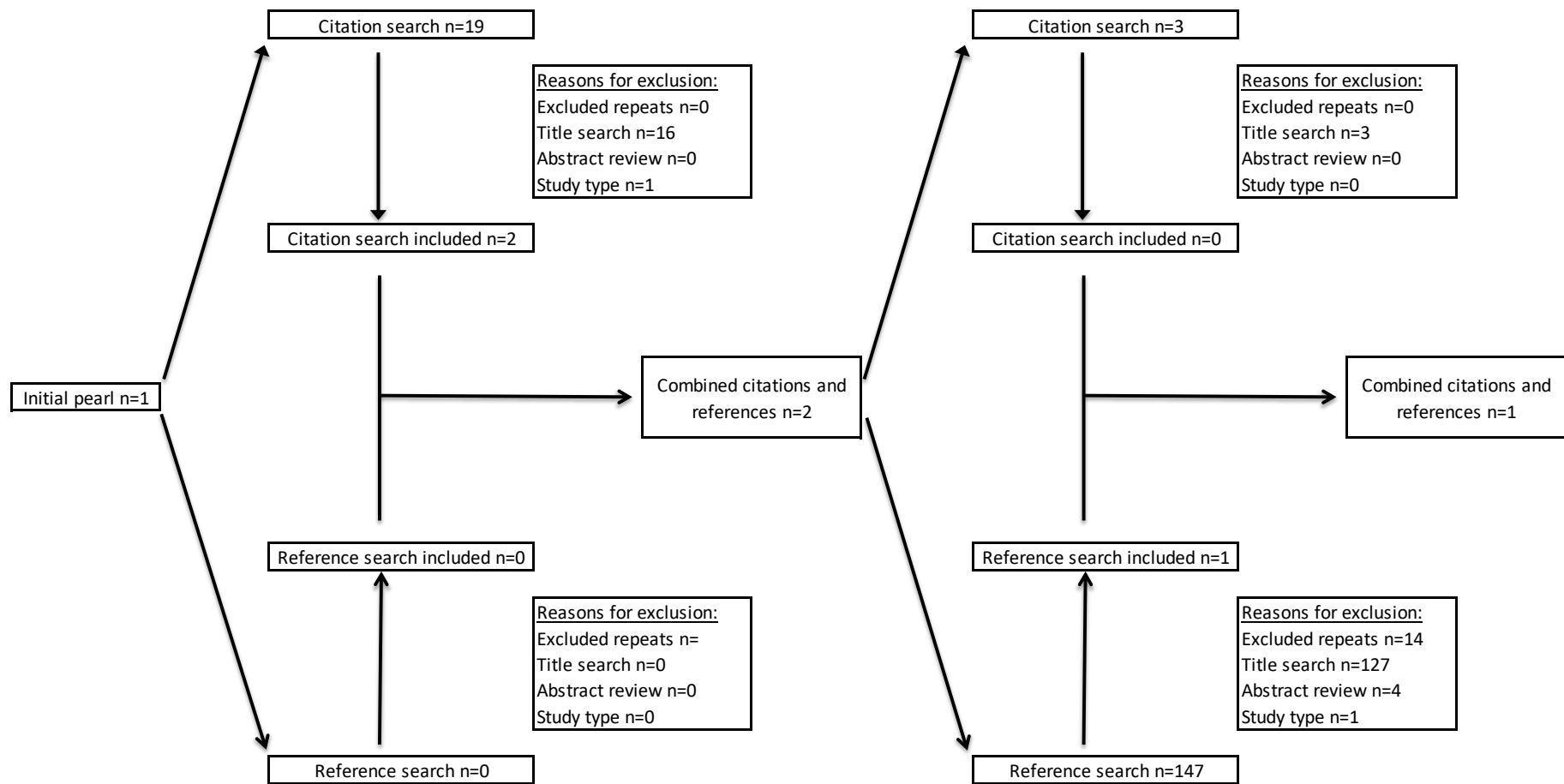

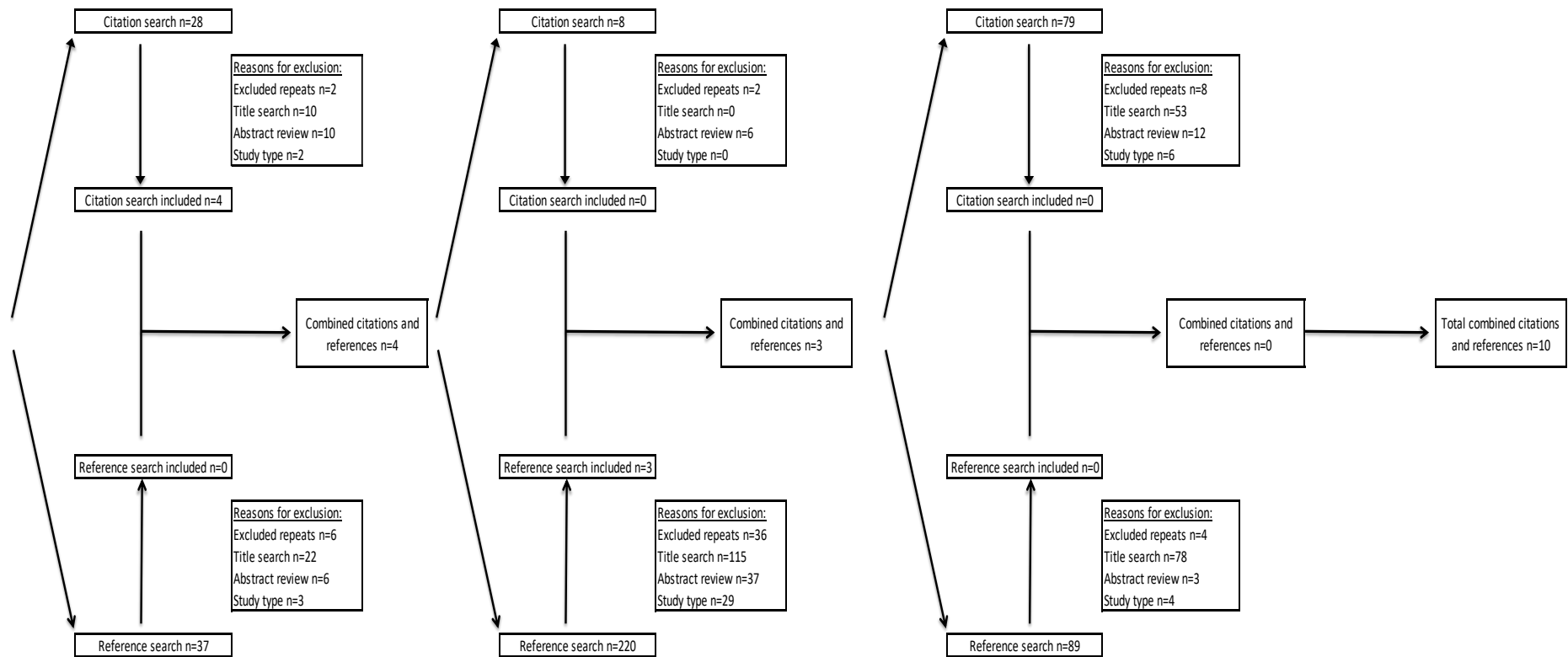

Figure A. 3: Flow diagram of the Pearl Growing Literature Review in the Web of Science (updated)

Supplement: Supplementary file 3 — Additional file 3: Fig. A.3. Flow diagram of the pearl-growing literature review in the Web of Science (updated). Flow diagram depicting the pearl-growing search strategy process with reasons for study exclusion. [file 12961_2024_1220_MOESM3_ESM.pdf]

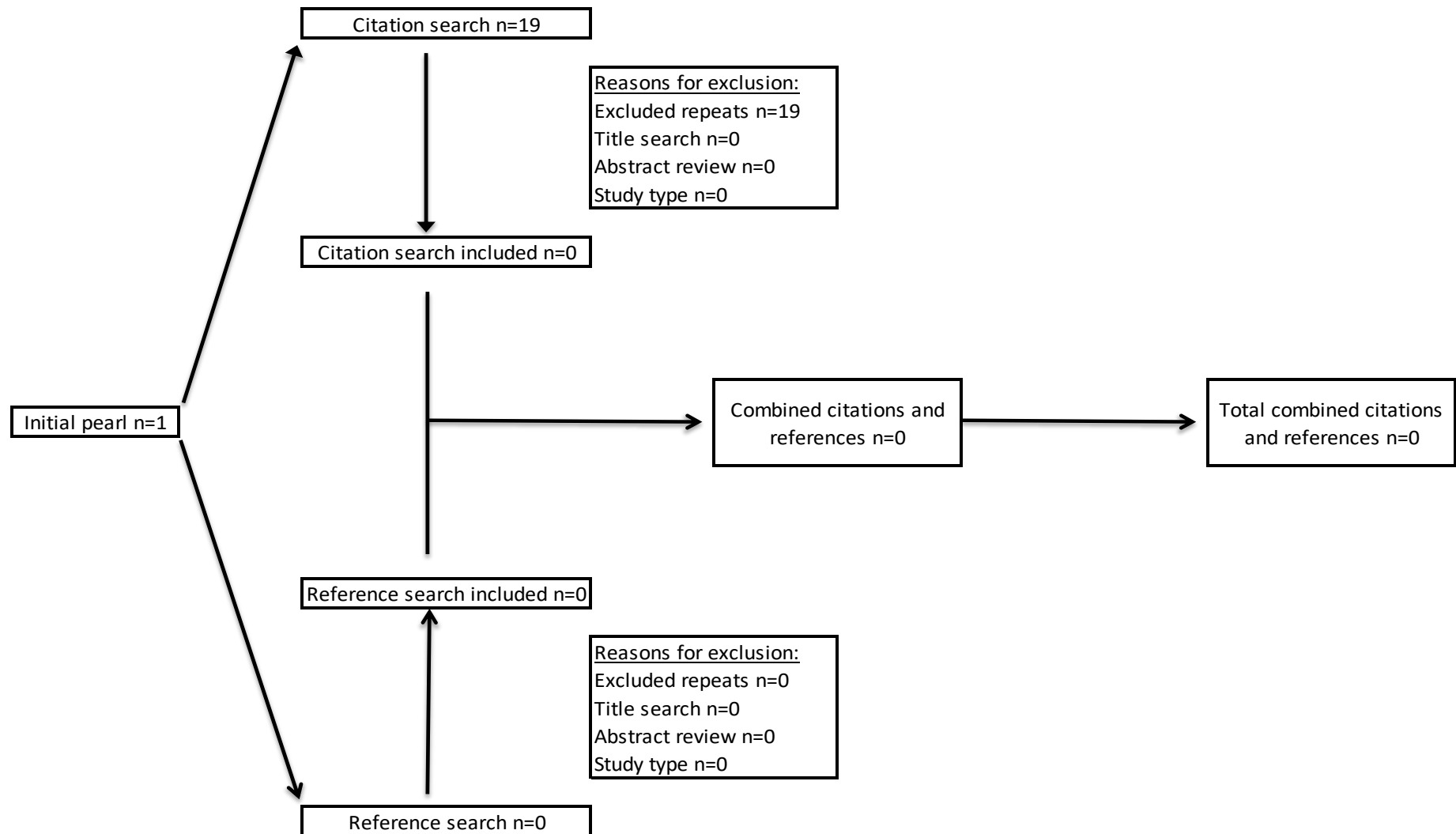

Figure A. 4: Flow diagram of the Pearl Growing Literature Review in SCOPUS (updated)

Supplement: Supplementary file 4 — Additional file 4: Fig. A.4. Flow diagram of the pearl-growing literature review in Scopus (updated). Flow diagram depicting the pearl-growing search strategy process with reasons for study exclusion. [file 12961_2024_1220_MOESM4_ESM.pdf]
